# *Leishmania braziliensis*: Strain-Specific Modulation of Phagosome Maturation

**DOI:** 10.3389/fcimb.2019.00319

**Published:** 2019-09-06

**Authors:** Tamara da Silva Vieira, Guillermo Arango Duque, Kévin Ory, Celia Maria Gontijo, Rodrigo Pedro Soares, Albert Descoteaux

**Affiliations:** ^1^Fundação Oswaldo Cruz - FIOCRUZ, Centro de Pesquisas René Rachou, Belo Horizonte, Brazil; ^2^INRS - Centre Armand-Frappier Santé Biotechnologie, Université du Québec, Laval, QC, Canada; ^3^Université de Rennes 1, CHU Rennes, INSERM, Rennes, France

**Keywords:** *Leishmania braziliensis*, virulence, lipophosphoglycan, GP63, macrophage, phagosome, intracellular survival

## Abstract

*Leishmania* (*Viannia*) *braziliensis* is responsible for the largest number of American tegumentary leishmaniasis (ATL) in Brazil. ATL can present several clinical forms including typical (TL) and atypical (AL) cutaneous and mucocutaneous (ML) lesions. To identify parasite and host factors potentially associated with these diverse clinical manifestations, we first surveyed the expression of two virulence-associated glycoconjugates, lipophosphoglycan (LPG) and the metalloprotease GP63 by a panel of promastigotes of Leishmania braziliensis *(L. braziliensis)* strains isolated from patients with different clinical manifestations of ATL and from the sand fly vector. We observed a diversity of expression patterns for both LPG and GP63, which may be related to strain-specific polymorphisms. Interestingly, we noted that GP63 activity varies from strain to strain, including the ability to cleave host cell molecules. We next evaluated the ability of promastigotes from these *L. braziliensis* strains to modulate phagolysosome biogenesis in bone marrow-derived macrophages (BMM), by assessing phagosomal recruitment of the lysosome-associated membrane protein 1 (LAMP-1) and intraphagosomal acidification. Whereas, three out of six *L. braziliensis* strains impaired the phagosomal recruitment of LAMP-1, only the ML strain inhibited phagosome acidification to the same extent as the *L. donovani* strain that was used as a positive control. While decreased phagosomal recruitment of LAMP-1 correlated with higher LPG levels, decreased phagosomal acidification correlated with higher GP63 levels. Finally, we observed that the ability to infect and replicate within host cells did not fully correlate with the inhibition of phagosome maturation. Collectively, our results revealed a diversity of strain-specific phenotypes among *L. braziliensis* isolates, consistent with the high genetic diversity within *Leishmania* populations.

## Introduction

The various species of the protozoan parasite *Leishmania* cause a spectrum of human diseases ranging from a relatively confined cutaneous lesion to a progressive and potentially fatal visceral infection (Alvar et al., 2012). Upon delivery in the vertebrate host by an infected sand fly, metacyclic *Leishmania* promastigotes are engulfed by phagocytes. To avoid destruction, these parasites have evolved efficient means of disarming the microbicidal functionality of their host cells (Arango Duque and Descoteaux, 2015; Podinovskaia and Descoteaux, 2015; Atayde et al., 2016; Martínez-López et al., 2018). To achieve this, infectious promastigotes rely on a panoply of virulence factors including two abundant components of their surface coat, the glycolipid lipophosphoglycan (LPG) and the GPI-anchored zinc metalloprotease GP63 (Moradin and Descoteaux, 2012; Olivier et al., 2012; Arango Duque and Descoteaux, 2015; Atayde et al., 2016). The use of mutants defective in either LPG or GP63 revealed that these molecules are indeed important for the colonization of phagocytic cells by promastigotes of *Leishmania donovani (L. donovani)* (Desjardins and Descoteaux, 1997; Lodge et al., 2006), *Leishmania major (L. major)* (Späth et al., 2000; Joshi et al., 2002), and *Leishmania infantum (L. infantum)* (Lázaro-Souza et al., 2018), all of which live in tight individual vacuoles. These virulence factors exert a profound impact on infected cells, altering signaling pathways (Descoteaux et al., 1991; Shio et al., 2012), inducing the production of inflammatory cytokines (Arango Duque et al., 2014), activating the inflammasome (de Carvalho et al., 2019), and inhibiting phagolysosomal biogenesis and functionality (Desjardins and Descoteaux, 1997; Späth et al., 2003; Lodge et al., 2006; Vinet et al., 2009; Matheoud et al., 2013; Matte et al., 2016). Of note, defective synthesis of LPG has no measurable effect on the ability of *Leishmania mexicana (L. mexicana)*, which lives in large communal vacuoles, to replicate in cultured macrophages and cause lesions in mice (Ilg, 2000; Ilg et al., 2001). These findings underline the fact that the relative contribution of a given virulence factor in the ability of promastigotes to colonize mammalian hosts varies among *Leishmania* species.

*Leishmania braziliensis* (subgenus *Viannia*) is responsible for the largest number of American tegumentary leishmaniasis cases in Brazil (ATL) (Alvar et al., 2012; PAHO/WH1O, 2017). ATL may exhibit several clinical forms including typical (TL), atypical (AL), and mucocutaneous (ML) lesions. TL may be confined at the bite site or metastasize to the oronasopharyngeal mucosa to give rise to ML. *L. braziliensis* AL lesions are scarce and they have been previously reported by Guimarães et al. in Bahia State (Guimarães et al., 2009) and by Quaresma et al. in the Minas Gerais State (Quaresma et al., 2018). Those lesions do not resemble classical TL lesions (round, ulcerated with elevated borders) and their ambiguous nature hinders correct diagnosis. Whether variations in GP63 and LPG levels are associated to the various clinical manifestations of ATL has not been investigated. In this regard, studies aimed at characterizing *GP63* in *L. braziliensis* revealed the presence of nearly 40 copies of this gene, as well as important sequence polymorphisms among clinical isolates (Medina et al., 2016). Characterization of LPG from *L. braziliensis* promastigotes revealed structural and compositional similarities to that of *L. donovani* (Soares et al., 2005), as well its strain-dependent capacity to induce inflammatory mediator release (Vieira Td et al., 2019).

To date, studies on the modulation of phagolysosome biogenesis by *Leishmania* promastigotes and on the contribution of LPG and GP63 to this process have focused mainly on species of the subgenus *Leishmania*. In the present study, we examined the levels of LPG and GP63 in a panel of *L. braziliensis* strains and surveyed their ability to interfere with phagosome maturation.

## Materials and Methods

### Ethics Statement

This study was carried out in accordance with the recommendations the Canadian Council on Animal Care on animal handling practices. Protocol 1706-07 was approved by the *Comité Institutionel de Protection des Animaux* of the INRS-Institut Armand-Frappier. *Leishmania braziliensis* field strains were obtained from patients living in the Xakriabá indigenous community located in São João das Missões municipality, Minas Gerais State, Brazil. Isolates from other endemic areas were obtained from the outpatient care facility at *Centro de Referência em Leishmanioses*—Instituto René Rachou/Fiocruz Minas from 1993 to 1998. Patient samples were obtained under informed consent procedures approved by the IRR Research Ethics Committee in Human Research, the National Committee for Research Ethics (*Comissão Nacional de Ética em Pesquisa*—CONEP) n° 355/2008, and the National Indian Foundation (*Fundação Nacional do Índio*—FUNAI) n° 149/CGEP/08.

### Cell Culture

Bone marrow-derived macrophages (BMM) were obtained from the bone marrow of 6–8 week-old female C57BL/6 mice and differentiated in complete DMEM [containing L-glutamine (Life Technologies), 10% v/v heat-inactivated fetal bovine serum (FBS) (Life Technologies), 10 mM HEPES (Bioshop) at pH 7.4, and penicillin-streptomycin (Life Technologies)] supplemented with 15% v/v L929 cell-conditioned medium (LCM) as a source of macrophage colony-stimulating factor. To render BMM quiescent prior to experiments, cells were transferred to tissue culture-treated plates containing glass coverslips for 16 h in complete DMEM without LCM (Descoteaux and Matlashewski, 1989). BMM were kept in a humidified 37°C incubator with 5% CO_2_.

Promastigotes were grown in *Leishmania* medium [M199-1X (Sigma) with 10% heat-inactivated FBS, 40 mM HEPES at pH 7.4, 100 μM hypoxanthine, 5 μM hemin, 3 μM biopterin, 1 μM biotin, MEM vitamin solution 1X, and penicillin-streptomycin] in a 26°C incubator (Soares et al., 2002; Arango Duque et al., 2014). The *L. braziliensis* strains used in this study (Table 1) include the World Health Organization reference strain (MHOM/BR/75/M2903), a ML isolate (MHOM/BR/1996/M15991), and a strain derived from *P. welcomei* sand flies (IWELL/BR/1981/M8401). The *L. braziliensis* RR051 strain was isolated from a TL lesion in the Minas Gerais State, and the RR418 (TL) and RR410 (AL) strains from lesions found in the Xakriabá community (Quaresma et al., 2018). These strains have been molecularly typed as *L. braziliensis* (Quaresma et al., 2018; Rugani et al., 2018)*. Leishmania donovani* LV9 and *L. major* Seidman A2 promastigotes were freshly differentiated from splenic or ear lesion-derived amastigotes, respectively.

**Table 1:** Leishmania strains used in this study.

**Strain/Isolate**	**Geographical origin**	**Lesion type**
MHOM/BR/75/M2903	Pará state, Brazil	TL
MHOM/BR/1995/RR051	Belo Horizonte, Minas Gerais State	TL
MHOM/BR/2009/RR418	São João das Missões, Minas Gerais State	TL
MHOM/BR/2008/RR410	São João das Missões, Minas Gerais State	AL
MHOM/BR/1996/M15991	Belém, Pará State	ML
IWELL/BR/1981/M8401	Belém, Pará State	N/A (strain isolated from vector)
MHOM/ET/67/Hu3:LV9	Ethiopia	Visceral
MHOM/SN/74 NIH clone A2 (A2WF)	Senegal	TL

### Infections and Phagosome Acidification Assays

Late stationary phase promastigotes (5-day cultures at > 50 × 10^6^ promastigotes/ml) from an early passage, or zymosan particles, were opsonised with serum from C5-deficient DBA/2 mice, resuspended in cold complete DMEM and fed to BMM (10:1 ratio) that had been seeded onto glass coverslips. Cells were incubated at 4°C for 5 min, and centrifuged for 2 min at 1,200 rpm (Arango Duque et al., 2013). Particle internalization was triggered by transferring cells to 37°C (Vinet et al., 2008; Arango Duque et al., 2014). Two hours post-internalization, infected macrophages were washed 3X with 1 ml warm DMEM to remove non-internalized promastigotes. Macrophages were either left at 37°C for an extra 22 h, or prepared for confocal microscopy. To assay phagosome acidification, BMM were incubated for 2 h with the acidotropic LysoTracker Red dye (diluted 1:1,000; Molecular probes) prior to the 2 h infection. In the case of the 24 h infection, infected macrophages were incubated in diluted LysoTracker for 2 h prior to the end of the infection time point. Cells were then washed and fixed.

For intracellular colonization assays, 6, 24, and 72 h-infected BMM seeded on coverlips were washed with PBS1X, stained with the Hema 3™ Stat Pack (Fisher), briefly washed with deionized water, and air-dried for 10 min. Coverslips were mounted onto a drop of Fluoromount-G and sealed. Images were acquired with a Qimaging camera (Teledyne Technologies International Corp) mounted on a Nikon Eclipse E800 microscope (60X objective). Images were compiled and analyzed with the ImageJ (Rueden et al., 2017) interphase of the Icy image analysis software (de Chaumont et al., 2012). Threshold segmentation was used to differentiate and enumerate BMM and intracellular *Leishmania* nuclei.

### Confocal Immunofluorescence Microscopy

Infected cells on coverslips were fixed with 2% paraformaldehyde (Thermo Scientific) for 20 min and blocked and permeabilized for 17 min with a solution of 0.1% Triton X-100, 1% BSA, 6% non-fat milk, 20% goat serum, and 50% FBS. This was followed by a 2 h incubation with a monoclonal rat antibody to Lysosome-associated membrane protein 1 (LAMP-1) (developed by J. T. August (1D4B) and purchased through the Developmental Studies Hybridoma Bank at the University of Iowa and the National Institute of Child Health and Human Development) diluted 1:200 in PBS1X. Subsequently, cells were incubated for 35 min in a solution containing an anti-rat antibody conjugated to Alexa-488 (diluted 1:500; Molecular Probes) and DAPI (1:40,000; Molecular Probes). Coverslips were washed three times with PBS1X after every step. After the final wash, coverslips were mounted cell-side facing a drop of Fluoromount-G (Southern Biotechnology Associates) that was placed on a glass slide (Fisher); coverslips were sealed with nail polish (Sally Hansen). Infected macrophages were imaged with the 63X objective of an LSM780 confocal microscope (Carl Zeiss Microimaging) and image processing was done with the ZEN 2012 software. In regards to LysoTracker-treated cells, fixed samples were incubated in diluted DAPI for 35 min prior to mounting. Recruitment was evaluated by scoring the presence of staining on the phagosome membrane (LAMP-1) and/or the phagosome lumen (LysoTracker) (Vinet et al., 2009; Arango Duque et al., 2014). One hundred phagosomes per coverslip were scored for every experimental condition, each done in duplicate.

### Electrophoresis, Western Blotting, and Zymography

Before lysis, stationary phase promastigotes, or infected BMM were washed with cold PBS1X containing 1 mM sodium orthovanadate and 5 mM 1,10-phenanthroline (Sigma). Pelleted parasites were resuspended in a lysis buffer containing 1% NP-40, 50 mM Tris-HCl (pH 7.5), 150 mM NaCl, 1 mM EDTA (pH 8), 15 mM 1,10-phenathroline and phosphatase and protease inhibitors (Roche). Samples were incubated at −70°C, sonicated with 20 s and thereafter centrifuged at 4°C for 15 min to remove insoluble material. After protein quantification, 30 μg of protein (10 μg in the case of promastigote lysate) was boiled (100°C) for 6 min in SDS sample buffer and migrated in SDS-PAGE gels. Proteins were transferred onto PDVF (phosphoglycan blots) or nitrocellulose membranes and thereafter blocked for 2 h in TBS1X-0.1% Tween containing 5% BSA. Membranes were subsequently probed (overnight at 4°C) with a mouse mAb (CA7AE; Cedarlane) that recognizes unbranched Galβ1,4Manα1-PO_4_ phosphoglycan moieties (Tolson et al., 1989), and detects *L. braziliensis* LPG (Vieira Td et al., 2019). Membranes were also probed for the presence of GP63 (mouse mAb #235 targeting the membrane-anchored version of *L. major* GP63) (Button et al., 1993; Macdonald et al., 1995); VAMP8 and syntaxin-5 (Stx5) (rabbit pAbs from Synaptic Systems). Anti-*L. donovani* aldolase (rabbit pAb, a kind gift from A. Jardim) and β-actin (Sigma) were used as loading controls. After washing, membranes were probed with suitable HRP-conjugated secondary antibodies for 1 h at room temperature, and incubated in ECL (GE Healthcare). Immunodetection was achieved via chemiluminescence (Arango Duque et al., 2014). To assess GP63 proteolysis via zymography, lysates were incubated at 50°C for 5 min in sample buffer without DTT, and then migrated in 10–12% SDS-PAGE gels containing 0.12% gelatin (Sigma) (Hassani et al., 2014; Arango Duque et al., 2019). Gels were incubated for 2 h in the presence of 50 mM Tris pH 7.4, 2.5% Triton X-100, 5 mM CaCl_2_ and 1 mM ZnCl_2_, followed by an overnight incubation at 37°C in a buffer containing 50 mM Tris pH 7.4, 5 mM CaCl_2_, 1 mM ZnCl_2_, and 0.01% NaN_3_. Protease activity was visualized by gradually distaining gels that were incubated in 0.5% Coomassie Brilliant blue (Sigma) for 1 h.

### Statistical Analysis

Statistical differences in recruitment levels were assessed using one-way ANOVA followed by Bonferroni *post-hoc* tests. Data were considered statistically significant when *p* < 0.05 and univariate column scatter graphs were constructed using GraphPad Prism 6.0 (GraphPad Software Inc).

## Results

### Expression of LPG and GP63 Varies Among Strains of *L. braziliensis*

**Figure 1:**
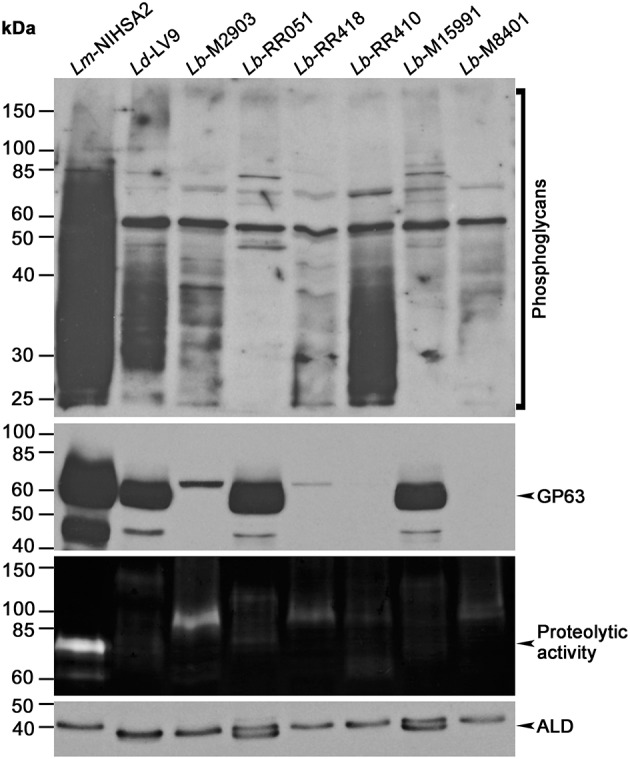
Expression of virulence-associated glycoconjugates in different *L. braziliensis* strains. *Leishmania* promastigote lysates were assayed for LPG, GP63, and aldolase expression by Western blot, and GP63 activity was probed via gelatin zymography. Images are representative of two independent experiments. *Lm, L. major; Ld, L. donovani; Lb, L. braziliensis; ALD, Ld aldolase*.

The ability of *Leishmania* promastigotes to colonize host cells and impair phagosome maturation and functionality is mediated to a large extent by the virulence factors LPG and GP63 (Chaudhuri et al., 1989; Späth et al., 2003; Moradin and Descoteaux, 2012; Atayde et al., 2016; Matte and Descoteaux, 2016). Here, we sought to determine the relative levels of LPG and GP63 expressed by promastigotes of a panel of *L. braziliensis* strains differing in their origin (Table 1). We included in our analysis *L. major* (NIH Seidman A2) and *L. donovani* (LV9) promastigotes as controls. Western blot analysis performed on promastigote lysates showed notable variations in the levels of LPG among the tested strains (Figure 1). Particularly, whereas the levels of LPG expressed by *L. braziliensis* RR410 were similar to those observed for *L. donovani* LV9, the levels detected in the other *L. braziliensis* strains were lower. In the case of GP63, we observed important differences among the *L. braziliensis* strains (Figure 1). Both *L. braziliensis* strains RR051 and M15991 expressed GP63 at levels comparable to those observed for *L. donovani* LV9. In contrast, GP63 levels were very low in the other strains. Interestingly, when we assessed the proteolytic activity of GP63 present in the *Leishmania* promastigotes lysates, we observed a lack of correlation with the GP63 levels detected by Western blot (Figure 1). Notably, *L. braziliensis* strains with low levels of GP63 (M2903 and RR418) showed high GP63 proteolytic activity, whereas *L. braziliensis* strains expressing higher GP63 levels (RR051 and M15991) showed reduced GP63 activity. These observations clearly demonstrated important intra-specific variations in the levels of detected LPG and GP63 (as well as GP63 activity) expressed by *L. braziliensis* strains isolated from patient with diverse ATL manifestations and from the insect vector.

### Cleavage of GP63 Substrates by *L. braziliensis* Strains

**Figure 2:**
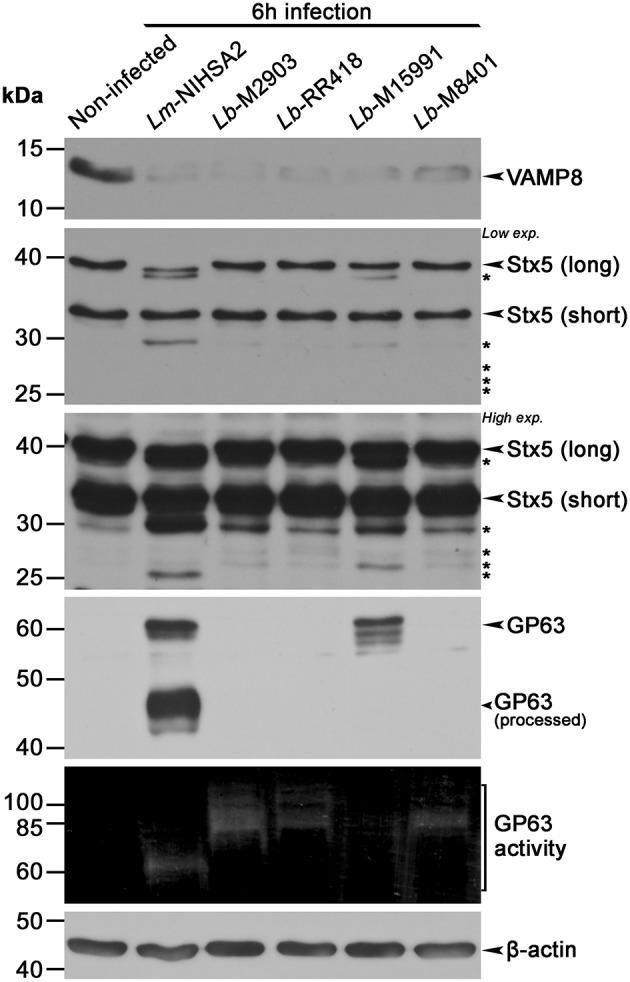
Proteolytic cleavage of phagosome-associated proteins by *Leishmania braziliensis (L. braziliensis)*. BMM were infected with opsonized stationary phase promastigotes from selected *L. braziliensis* strains, and the cleavage of phagosomal proteins VAMP8 and Stx5 was assessed via Western blot. GP63 activity was also assayed via gelatin zymography and β-actin was used as loading control. The long form of Stx5 is localized at the Golgi and ER, and the short one at the Golgi. Asterisks denote cleavage fragments. *Lm, L. major*; *Lb, L. braziliensis*.

Given the variations in GP63 levels and activity observed among the *L. braziliensis* isolates, we investigated the impact of these differences on the cleavage of phagosomal host cell proteins known to be targeted by GP63 (Matheoud et al., 2013). To this end, we performed Western blot analyses to assess the levels and integrity of the soluble *N*-ethylmaleimide-sensitive-factor attachment protein receptors (SNAREs) VAMP8 and Stx5, in lysates of BMM infected for 6 h with promastigotes of selected *L. braziliensis* strains (M2903, RR418, M15991, and M8401) and promastigotes of *L. major* NIHS A2 as control. As shown in Figure 2, VAMP8 was cleaved to the same extent by all *L. braziliensis* strains and by *L. major* NIHS A2, regardless of the levels and activity of GP63 detected in the cell lysates. In contrast, cleavage of the endoplasmic reticulum (ER)- and Golgi-resident SNARE Stx5 was strain-dependent and did not entirely correlate with the levels and activity of GP63 detected in the cell lysates (Figure 2). Collectively, these results indicate that cleavage of host cell GP63 substrates occurs in BMM infected with all *L. braziliensis* strains tested, albeit with some differences in the extent of cleavage. These findings also suggest that sensitivity to GP63 cleavage is substrate-specific.

### *L. braziliensis* Impairs Phagosomal Recruitment of LAMP-1 in a Strain-Specific Manner

**Figure 3:**
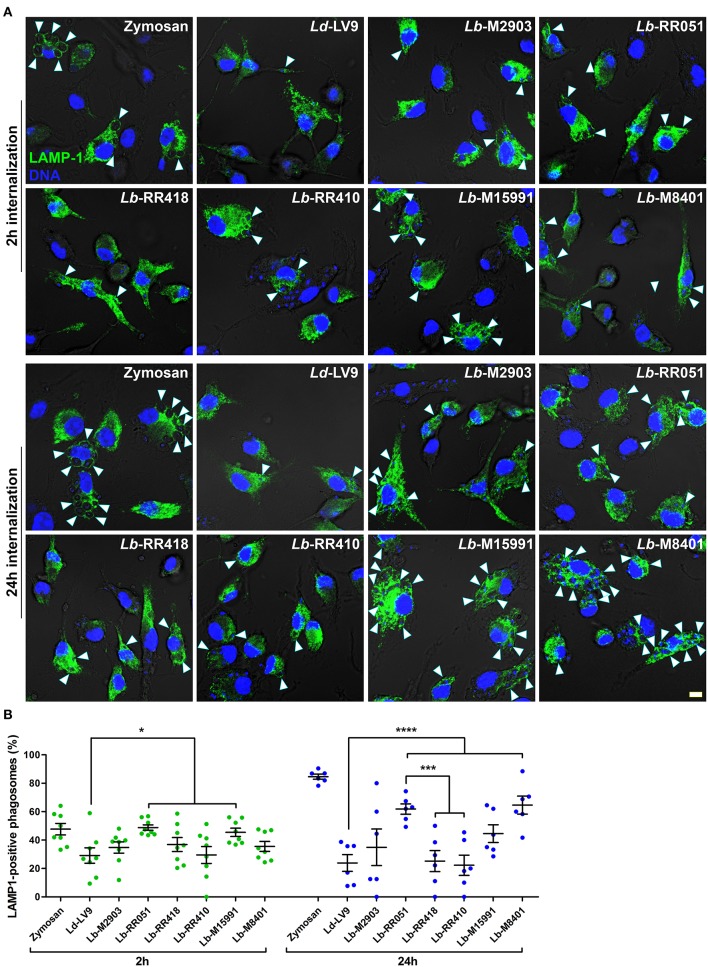
LAMP-1 recruitment to parasitophorous vacuoles harboring *L. braziliensis* parasites. **(A)** BMM were infected with opsonized stationary phase promastigotes from different *L. braziliensis* strains, and LAMP-1 (green) recruitment to parasite-containing phagosomes was visualized via immunofluorescence at 2 and 24 h post-internalization. DNA is shown in blue; scale bar = 5 μm. White arrowheads indicate LAMP1 recruitment. **(B)** Quantification of LAMP-1-positive phagosomes at 2 h (green dots) and 24 h (blue dots) post-phagocytosis. Bars depict the mean ± SEM of % positive phagosomes counted over three independent experiments done at least in duplicate coverslips. *p < 0.05; ***p < 0.001; ****p < 0.0001. *Ld, L. donovani*; *Lb, L. braziliensis*.

Given the variations observed among our panel of *L. braziliensis* strains in LPG and GP63 levels and activity, as well as substrate cleavage, we investigated the impact of *L. braziliensis* promastigotes on phagosome maturation. To this end, we incubated BMM with promastigotes from our panel of *L. braziliensis* strains for 2 and 24 h and assessed the recruitment of the lysosomal marker LAMP-1 to phagosomes. Promastigotes of *L. donovani* (LV9 strain), which efficiently inhibit phagosome maturation and phagosomal recruitment of LAMP-1 (Scianimanico et al., 1999), and zymosan were used as controls. At 2 h after the initiation of phagocytosis, we observed a higher recruitment of LAMP-1 to phagosomes containing *L. braziliensis* strains RR051 and M15991 compared to phagosomes containing *L. donovani* LV9 (Figures 3A,B). As expected, recruitment of LAMP-1 to phagosomes containing zymosan was higher to that observed for phagosomes induced by promastigotes of *L. donovani* LV9 and of those from *L. braziliensis* isolated from an AL lesion (RR410) (Figure 3). At 24 h post-infection, the presence of LAMP-1 on phagosomes harboring *L. donovani* promastigotes LV9 remained very low, as was also the case for phagosomes containing *L. braziliensis* M2903, RR410, and RR418. However, recruitment of LAMP-1 to phagosomes harboring *L. braziliensis* RR051 and M8401 was significantly higher than the levels observed for phagosomes containing the other *L. braziliensis* strains and *L. donovani* LV9 (Figure 3). These results suggest that the ability *L. braziliensis* promastigotes to interfere with phagosome maturation varies among strains.

### Phagosome Acidification Is Differentially Modulated by *L. braziliensis* Strains

**Figure 4:**
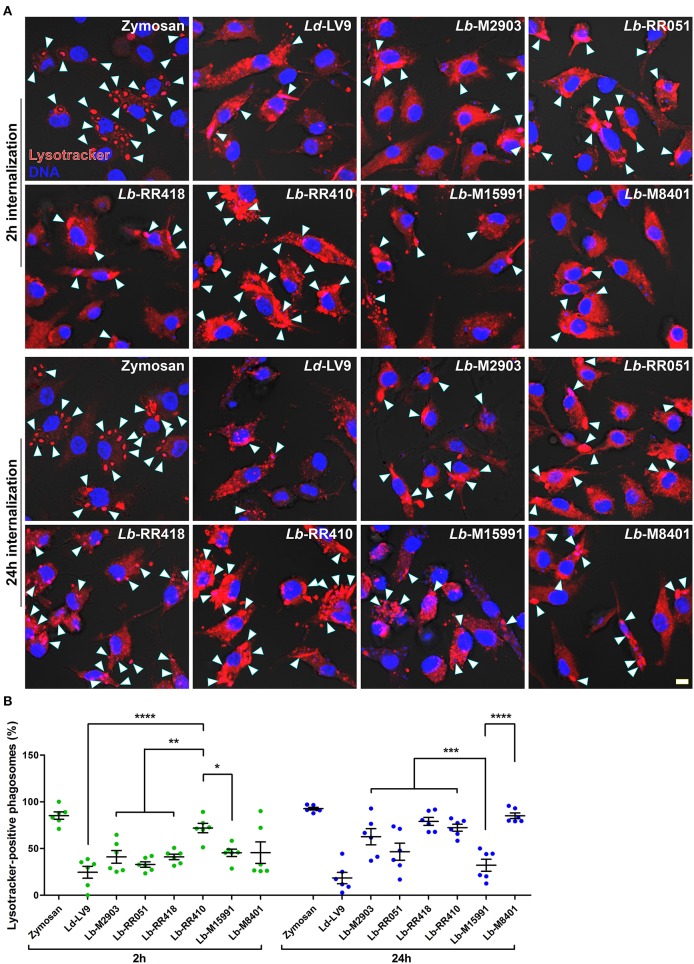
Acidification of parasitophorous vacuoles harboring *L. braziliensis* parasites. **(A)** Bone marrow-derived macrophages (BMM) were infected with opsonized stationary phase promastigotes from different *L. braziliensis* strains, and acidification of parasite-containing vacuoles was assayed via LysoTracker staining (red) at 2 and 24 h post-internalization. DNA is shown in blue; scale bar = 5 μm. White arrowheads indicate LysoTracker-positive phagosomes. **(B)** Quantification of LysoTracker-positive phagosomes at 2 h (white bars) and 24 h (gray bars) post-phagocytosis. Bars depict the mean ± SEM of % positive phagosomes counted over three independent experiments done in duplicate. *p < 0.05; **p < 0.01; ***p < 0.001; ****p < 0.0001. *Ld, L. donovani*; *Lb, L. braziliensis*.

Consistent with their ability to inhibit phagolysosome biogenesis (Desjardins and Descoteaux, 1997), we previously reported that *L. donovani* promastigotes efficiently impair phagosome acidification (Vinet et al., 2009). To further characterize the impact of *L. braziliensis* promastigotes on phagosome maturation, we used the lysotropic dye LysoTracker Red to monitor acidification kinetics of phagosomes harboring the various *L. braziliensis* strains. Consistent with previous studies, at 2 h post-infection, acidification occurred in the majority of zymosan-harboring phagosomes but was hindered in phagosomes containing *L. donovani* LV9 promastigotes (Figures 4A,B). Similar impairment of phagosome acidification was observed for all *L. braziliensis* strains, with the exception of the strain isolated from an AL lesion (RR410) (Figures 4A,B). At 24 h post-infection, most phagosomes harboring *L. donovani* LV9 (80%) and the *L. braziliensis* ML isolate (M15991) (70%) remained negative for LysoTracker Red (Figure 4). In contrast, over 70% of phagosomes containing *L. braziliensis* isolates RR418 and RR410 were positive for LysoTracker Red at 24 h (Figure 4B). These data indicate that most *L. braziliensis* strains in our panel inhibit phagosome acidification during the early phase of macrophage infection. However, at later time points, the capacity to hinder phagosome acidification varies in a strain-specific manner.

### Colonization of Macrophages by *L. braziliensis* Strains Does Not Fully Correlate With the Ability to Inhibit Phagosome Maturation and Acidification

**Figure 5:**
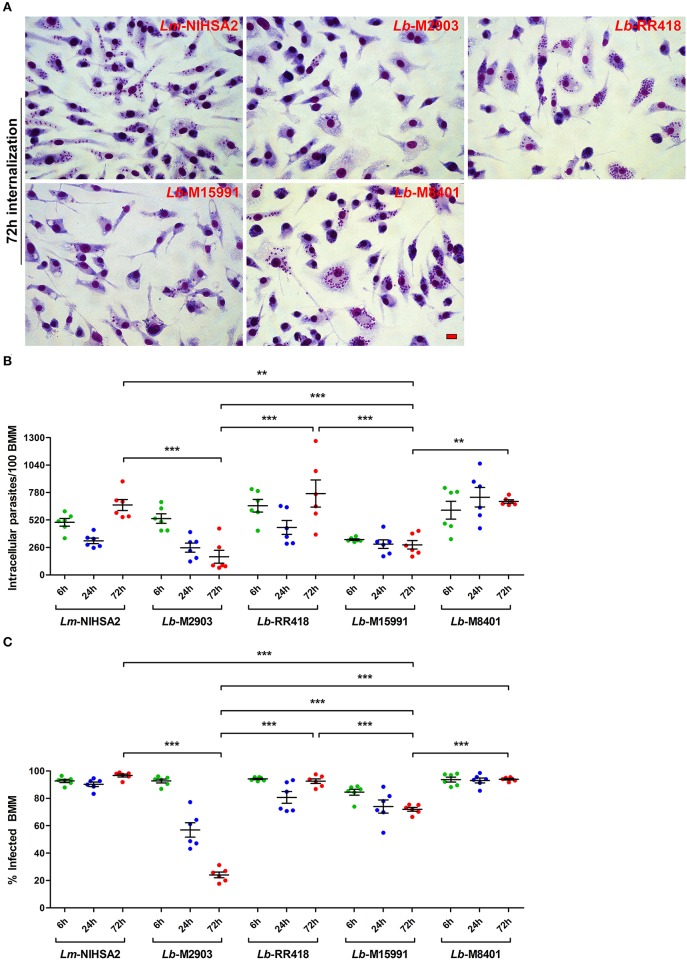
Intracellular survival and replication of *L. braziliensis* parasites. BMM were infected with opsonized stationary phase promastigotes from selected *L. braziliensis* strains, and intracellular survival was quantified in Hema 3-stained cells. **(A)** Representative images of the 72 h time point. Scale bar = 10 μm. Intracellular survival at 6 (green dots), 24 (blue dots), and 72 h (red dots) post-infection was assessed via the quantification of internalized parasites in 100 macrophages **(B)** and the percentage of infected cells **(C)**. In both **(B,C)**, bars depict the mean ± SEM of two independent experiments done in triplicate coverslips. **p < 0.01; ***p < 0.001. *Lm, L. major*; *Lb, L. braziliensis*.

Previous studies with *L. donovani* and *L. major* (Desjardins and Descoteaux, 1997; Späth et al., 2003; Vinet et al., 2009) revealed a correlation between the ability of these parasites to impair phagosome maturation and the ability to colonize macrophages. To investigate whether such a correlation exists for the *L. braziliensis* strains under study, we incubated BMM for 2 h with promastigotes of selected strains (M2903, RR418, M15991, and M8401) and promastigotes of *L. major* NIHS A2 as control. We then quantified the number of parasites per 100 macrophages and the percentage of infected macrophages at 6, 24, and 72 h post-phagocytosis. As shown in Figure 5, the ability to survive and replicate over time within BMM varied among the *L. braziliensis* strains analyzed. At the exception of strain M2903, which displayed reduced ability to survive in BMM over 72 h, all the other strains persisted and two of them (RR418 and M8401) replicated as was the case for *L. major* NIHS A2. Interestingly, *L. braziliensis* strain M2903, which survived poorly in BMM, was among the most efficient strains at inhibiting phagosome maturation (Figures 3, 4). For strains RR418 and M8401, their ability to replicate in BMM correlated with their capacity to impair the phagosomal recruitment of LAMP-1 and acidification during the early phases of infection (Figures 3, 4). These data are consistent with the notion that factor(s) other than the capacity to impair phagosome maturation are required for colonization of host cells by *L. braziliensis*.

## Discussion

The *Leishmania* virulence factors LPG and GP63 contribute to the ability of promastigotes to colonize phagocytic cells by targeting key host cell host defense mechanisms, including the biogenesis of microbicidal phagolysosomes. In the present study, we sought to examine the levels of LPG and GP63 expressed by promastigotes of *L. braziliensis* (subgenus *Viannia*) strains isolated from patients exhibiting various clinical manifestations of ATL and from the insect vector. We also characterized the ability of these *L. braziliensis* strains to impair phagosome maturation and to infect and replicate within macrophages.

Our results revealed an unexpected diversity of expression patterns for both LPG and GP63 among the evaluated *L. braziliensis* strains. Although some strains expressed LPG levels similar to those of *L. donovani* LV9 promastigotes, other strains expressed very low LPG levels. Similarly, some *L. braziliensi*s strains expressed GP63 levels comparable to those observed in *L. donovani* LV9, whereas other strains expressed very low GP63 levels. Interestingly, we noted that GP63 activity varies from strain to strain, and does not correlate with GP63 levels detected by Western blot. Whether the polymorphims detected in the *GP63* genes of *L. braziliensis* (Medina et al., 2016) affected the recognition of GP63 by our anti-GP63 antibody is however unclear. Clearly, the significance of these observations deserves to be further investigated.

As part of their strategy to colonize host phagocytes, *Leishmania* promastigotes alter the composition and properties of the parasitophorous vacuole (Moradin and Descoteaux, 2012; Séguin and Descoteaux, 2016). Phagosomal recruitment of the lysosomal protein LAMP-1 is a widely used marker of phagosome maturation (Huynh et al., 2007). In the case of *Leishmania* promastigotes, delayed phagosomal acquisition of LAMP-1 following phagocytosis supported the notion that these parasites impair phagolysosomal biogenesis (Scianimanico et al., 1999; Lerm et al., 2006; Verma et al., 2017). Interestingly, we found that the ability to inhibit the phagosomal recruitment of LAMP-1 varies significantly among our panel of *L. braziliensis* strains. Phagosome acidification is an important consequence of the maturation process and we previously reported that it is efficiently inhibited by *L. donovani* promastigotes (Vinet et al., 2009). Similar to the recruitment of LAMP-1, we observed an important variation among promastigotes of the *L. braziliensis* strains tested in their capacity to inhibit phagosomal acidification. Interestingly, whereas promastigotes of *L. donovani* LV9 efficiently inhibited both phagosome acidification and recruitment of LAMP-1, we observed no correlation between the ability to inhibit phagosomal recruitment of LAMP-1 and phagosome acidification among the *L. braziliensis* strains. Previous work from our group revealed that acquisition of LAMP-1 and of the v-ATPase by phagosomes occurs through two distinct mechanisms (Vinet et al., 2009). In the case of *L. donovani*, LPG is the molecule responsible for inhibiting both the phagosomal recruitment of LAMP-1 and acidification (Scianimanico et al., 1999; Vinet et al., 2009). However, the ability of *L. braziliensis* strains to interfere with phagosome maturation does not appear to correlate with LPG levels.

In addition to LPG, *Leishmania* promastigotes use the metalloprotease GP63 to modulate the composition and function of phagosomes through the cleavage of host proteins such as VAMP3, VAMP8, and Synaptotagmin XI (Matheoud et al., 2013; Arango Duque et al., 2014; Casgrain et al., 2016; Matte and Descoteaux, 2016; Matte et al., 2016). Since VAMP8 is required for antigen cross-presentation (Matheoud et al., 2013), its cleavage by the various *L. braziliensis* strains suggests that they efficiently inhibit antigen cross-presentation. Future experiments will specifically address this issue. On the other hand, the endoplasmic reticulum- and Golgi-resident SNARE Stx5 is partially cleaved, to varying extents, by our *L. braziliensis* strains. This SNARE regulates trafficking between the phagosome and the secretory pathway (Cebrian et al., 2011; Arango Duque et al., 2019) and contributes to the expansion of communal parasitophorous vacuoles harboring *L. amazonensis* (Canton and Kima, 2012). The significance of its cleavage by *L. braziliensis* for establishment and replication within macrophages is an issue that will deserve further investigation.

In *L. braziliensis, GP63* is present on chromosome 10 and strains isolated from different clinical manifestations from the same geographical region have conserved domains and display specific polymorphisms in their catalytic sites (Medina et al., 2016; Sutter et al., 2017; Quaresma et al., 2018). This variability could result in different virulence patterns and clinical outcomes. Of interest, a recent genomic analysis of *Leishmania* clinical isolates revealed important differences among genetically highly related *Leishmania* strains, including both in amplification and in loss of genes linked to parasite infectivity such as *GP63* (Bussotti et al., 2018). Whether the diversity of GP63 levels and activity portrayed by the *L. braziliensis* strains is the consequence of gene amplification associated to environmental adaptation is a likely possibility that deserves further investigation. Similar to LPG and GP63, GIPLs are highly expressed on the *Leishmania* surface (Assis et al., 2012). They are inhibitory molecules impairing NO and cytokine production by murine macrophages and their role on phagosome maturation and intracellular survival will be assayed in prospective studies. Together, our findings underline the importance of performing functional genetic analyses with these clinical *L. braziliensis* strains to directly assess the importance of LPG and GP63 in the colonization of host phagocytes, and ultimately in the pathogenesis of ATL.

For the past several decades, research on virulence or immune subversion mechanisms of *Leishmania* has been for the most part performed with reference or laboratory strains. Results obtained with those strains allowed for the discovery of several biological processes. For instance, the Th1/Th2 dichotomy and the importance of IL-4 in mediating susceptibility to infection were discovered using a particular *L. major* strain (Heinzel et al., 1989). However, studies using other *L. major* strains led to opposite results (Noben-Trauth et al., 1996, 1999). In the case of *L. braziliensis* ATL strains, our study revealed an unexpected diversity in terms of expression of virulence molecules and ability to interfere with phagosome maturation. Clearly, these studies highlight the fact that it is important to exert caution when drawing broad conclusions based on observations obtained with a single strain or isolate of a given *Leishmania* species.

## Data Availability

All datasets generated for this study are included in the manuscript and/or the supplementary files.

## Ethics Statement

Mice were manipulated under the guidelines of protocol 1706-07 of the Comité Institutionel de Protection des Animaux of the INRS-Institut Armand-Frappier, which respects animal handling practices promulgated by the Canadian Council on Animal Care. *Leishmania braziliensis* field strains were obtained from patients living in the Xakriabá indigenous community located in São João das Missões municipality, Minas Gerais State, Brazil. Isolates from other endemic areas were obtained from the outpatient care facility at Centro de Referência em Leishmanioses—Instituto René Rachou/Fiocruz Minas from 1993 to 1998. Patient samples were obtained under informed consent procedures approved by the IRR Research Ethics Committee in Human Research, the National Committee for Research Ethics (Comissão Nacional de Ética em Pesquisa—CONEP) n° 355/2008, and the National Indian Foundation (Fundação Nacional dÍo ndio—FUNAI) n° 149/CGEP/08.

## Author Contributions

GA, TS, RS, and AD conceived and designed the study, contributed to the data analysis, and drafted and revised the manuscript. GA, TS, and KO performed the experiments. CG provided the *L. braziliensis* strains. GA, TS, RS, and AD wrote and revised the manuscript. All authors read and approved the final version of this manuscript.

### Conflict of Interest Statement

The authors declare that the research was conducted in the absence of any commercial or financial relationships that could be construed as a potential conflict of interest.
